# Sperm motility of oysters from distinct populations differs in response to ocean acidification and freshening

**DOI:** 10.1038/s41598-019-44321-0

**Published:** 2019-05-28

**Authors:** Laura J. Falkenberg, Craig A. Styan, Jon N. Havenhand

**Affiliations:** 1 Faculty of Engineering Sciences, University College London, Adelaide, South Australia 5001 Australia; 2 Present Address: Simon F.S. Li Marine Science Laboratory, School of Life Sciences, The Chinese University of Hong Kong, Hong Kong, SAR China; 3 Department of Marine Sciences, Tjärnö Marine Laboratory, University of Gothenburg, 45296 Strömstad, Sweden

**Keywords:** Animal physiology, Climate-change ecology

## Abstract

Species’ responses to climate change will reflect variability in the effects of physiological selection that future conditions impose. Here, we considered the effects of ocean acidification (increases in *p*CO_2_; 606, 925, 1250 µatm) and freshening (reductions in salinity; 33, 23, 13 PSU) on sperm motility in oysters (*Crassostrea gigas*) from two populations (one recently invaded, one established for 60+ years). Freshening reduced sperm motility in the established population, but this was offset by a positive effect of acidification. Freshening also reduced sperm motility in the recently invaded population, but acidification had no effect. Response direction, strength, and variance differed among individuals within each population. For the established population, freshening increased variance in sperm motility, and exposure to both acidification and freshening modified the performance rank of males (i.e. rank motility of sperm). In contrast, for the recently invaded population, freshening caused a smaller change in variance, and male performance rank was broadly consistent across treatments. That inter-population differences in response may be related to environmental history (recently invaded, or established), indicates this could influence scope for selection and adaptation. These results highlight the need to consider variation within and among population responses to forecast effects of multiple environmental change drivers.

## Introduction

Species’ responses to climate change vary among levels of organisation. For example, differences have been found among Phyla^1^, among populations of the same species^2–4^, and among individuals of the same populations^5–7^. Where variation in responses is large and heritable there is greater scope for selection^8,9^ and, thereby, the potential for fitness to be influenced^10,11^. Consequently, understanding response variability among individuals is fundamental to identifying the potential for adaptation, and improving our ability to predict impacts of future change^3^.

Strong selective pressures on reproduction can carry over into subsequent life stages. For example, sperm selection plays a key role in shaping the subsequent generation in many broadcast-spawning marine organisms^12,13^. Moreover, gametes of broadcast-spawning organisms are often particularly sensitive to selection as they are released into the environment and typically possess limited buffering capacities^8,14,15^. Where environmental conditions modify gamete motility, selection may act upon individuals to result in the modification, or loss, of certain populations^16^.

Ongoing ocean acidification will likely generate significant novel selection pressures. While many studies have indicated that ocean acidification could lead to reduced sperm motility^8,17–19^, others have found it can remain unchanged^20^, or even increase^21^. Yet ocean acidification does not operate in isolation, and many studies show that multiple simultaneous drivers can have very different effects to those of drivers operating alone^22^. Climate projections indicate than in many regions freshening – the decline in seawater salinity – will arise from altered patterns of precipitation and ice sheet melt^23–25^. When experienced in isolation, freshening is reported to lower sperm motility in a range of species (e.g. Atlantic cod *Gadus morhua*^26^, polychaete *Galeolaria caespitosa,* and Pacific oyster *Crassostrea gigas*^27^). However, the combined effects of freshening and ocean acidification on sperm motility remain unassessed.

We, therefore, measured the effects of ocean acidification and freshening on sperm motility at both the population and individual levels. To study these effects we used the model oyster species *C. gigas*. *C. gigas* is an important aquaculture and invasive species^28^, whose persistence into the future may be modified as its reproduction can be influenced by both ocean acidification^29^ and salinity^30^. Here, we tested: (1) the effects of ocean acidification and freshening on sperm motility; (2) if (and how) these effects varied between populations and among individuals; and (3) the impacts of acidification and freshening on male performance rank. We investigated these responses in *C. gigas* individuals sampled from two populations: one that invaded the low-salinity west coast of Sweden recently (within ≤10 years of this study; termed “invasive”), and one that has been established for 60+ years in the stable full-salinity waters of Guernsey, British Channel Islands (termed “established”). This work contributes to developing a broader understanding of the extent to which populations, and male performance broadly, will be modified in the future.

## Results

**Figure 1:**
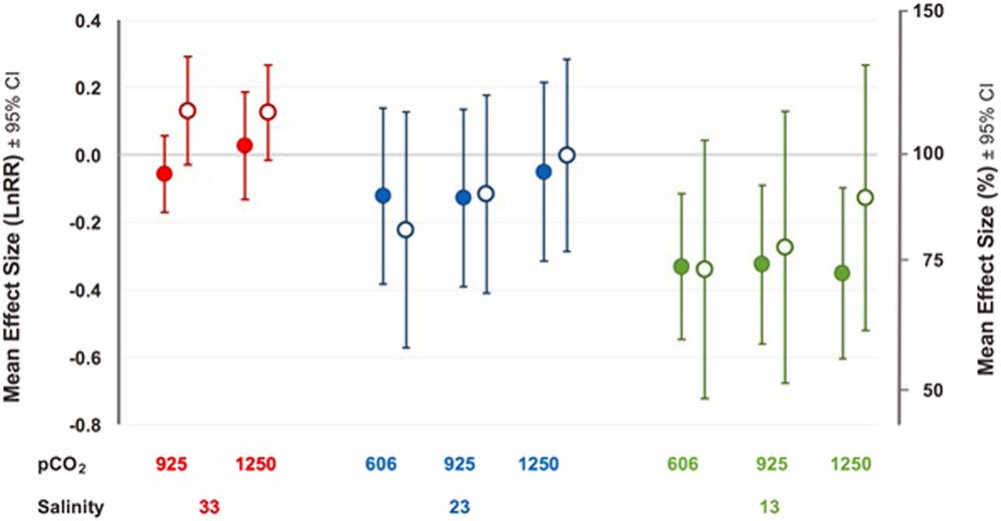
Mean effects (mean log_e_ response ratio, LnRR, ±95% CI) of ocean acidification (*p*CO_2_, µatm) and freshening (salinity, PSU) on sperm motility (measured in terms of Sperm Accumulated At Surface, SAAS) in oysters from two populations (invasive, Sweden, closed circles and established, Guernsey, open circles; *n* = 14 per population). LnRR’s calculated using 606 µatm CO_2_ × 33 PSU as reference. Note difference in scale on y-axis from Fig. 2.

Across populations, sperm motility declined with freshening (Fig. 1). Responses to acidification, however, differed; sperm motility increased with acidification in males from the established population, whereas sperm motility did not change substantively with acidification for those from the recently invaded population (open *vs* closed circles respectively, Fig. 1). Consequently, for established population males, the positive effects of acidification offset the negative effects of freshening such that their combination resulted in sperm motility that was equivalent to that in control conditions (i.e. LnRR ≈ 0; Fig. 1). Similar antagonistic effects of freshening and acidification were not seen in males from the recently invaded population, which therefore showed freshening-induced reductions in sperm motility under future climate scenarios. These findings were reflected in the formal PERMANOVA results (Table 1), which showed that the two populations responded differently to *p*CO_2_ (Population × *p*CO_2_, *p* = < 0.01), and that responses to *p*CO_2_ and freshening varied significantly among individuals (Salinity × *p*CO_2_ × Individual[Population], *p* = < 0.01).

**Table 1:** ANOVA on Sperm Accumulated At Surface (SAAS) of individual oysters from two populations (invasive, Sweden; established, Guernsey) exposed to ocean acidification (*p*CO_2_) and freshening (salinity).

Source	df	MS	F	*p*
Population	1	303.67	2.11	0.14
Salinity	2	114.28	3.86	**0.02**
*p*CO_2_	2	17.00	11.05	**<0.01**
Individual(Population)	26	144.04	106.51	**<0.01**
Population × Salinity	2	12.64	0.43	0.65
Population × *p*CO_2_	2	13.07	8.50	**<0.01**
Salinity × *p*CO_2_	4	0.71	0.34	0.84
Salinity × Individual(Population)	52	29.62	21.90	**<0.01**
*p*CO_2_ × Individual(Population)	52	1.54	1.14	0.25
Population × Salinity × *p*CO_2_	4	2.13	1.03	0.40
Salinity × *p*CO_2_ × Individual(Population)	104	2.06	1.53	**<0.01**
Residual	504	1.35		

*p* ≤ 0.05 shown in bold.

**Figure 2:**
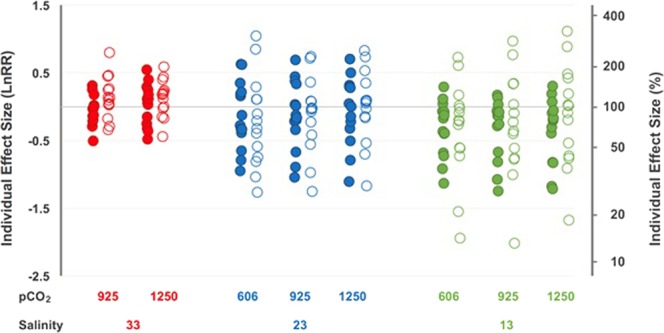
Individual effects (log_e_ response ratio, LnRR) of ocean acidification (*p*CO_2_, µatm) and freshening (salinity, PSU) on sperm motility (measured in terms of Sperm Accumulated At Surface, SAAS) in oysters from two populations (invasive, closed circles and established, open circles). LnRR’s calculated using 606 µatm CO_2_ × 33 PSU as reference. Note difference in scale on y-axis from Fig. 1.

Sperm from different males responded differently to the treatments in terms of their motility; some males showed improvements in sperm motility under acidification and freshening, whereas others were negatively affected (Fig. 2). Freshening caused a clear increase in response variance among males, especially in the established population (Fig. 2). In contrast, acidification had no effect on response variance.

**Figure 3:**
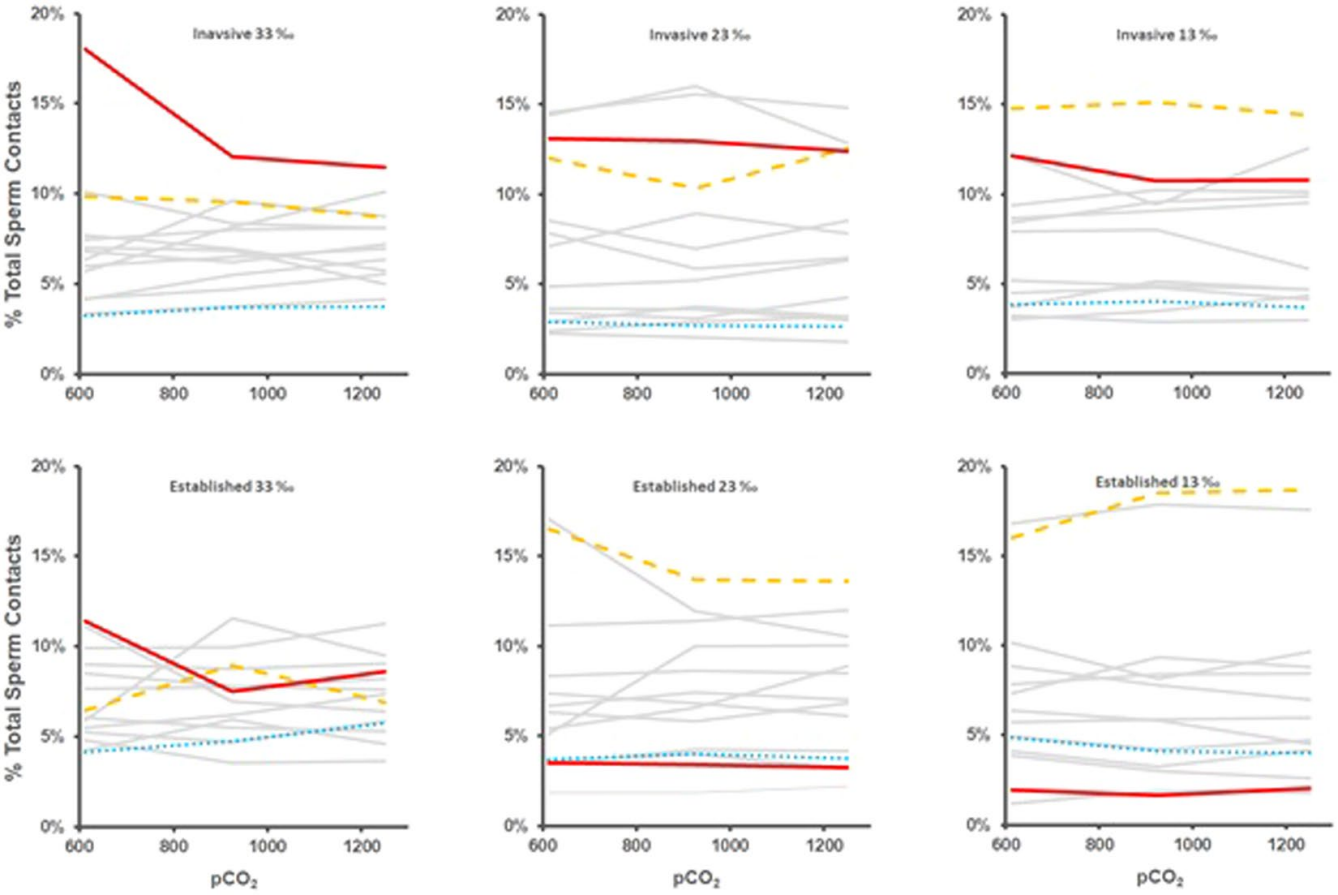
Change in male performance rank with ocean acidification (*p*CO_2_, µatm) and freshening (salinity, PSU). Data are sperm contacts for each male as a percentage of total number of sperm contacts (sum of all contacts for all males) in each treatment. Lines represent individual males. For each population: the highest performing male in the ambient (control) treatment (606 µatm CO_2_, 33‰) is indicated by a solid/red line; the lowest performing male in the control treatment is indicated by a dotted/blue line; the highest performing male in the most extreme treatment (1250 µatm CO_2_, 13‰) is indicated by a dashed/orange line.

Differential sensitivity of males to acidification and freshening was reflected in relative male performance, which was calculated for each treatment as the percentage of total sperm contacts (SAAS) for that population that were obtained by any given individual (Fig. 3). In the recently invaded population, males that performed best (i.e. had a high performance rank) in control conditions tended to retain performance rank under acidification and freshening (e.g. small changes in performance rank in solid-red, dotted-blue, and dashed-yellow lines in Fig. 3, upper panels). In the established population, however, the highest ranked male under control conditions was one of the poorest-performing males when exposed to acidification and freshening, and *vice versa* (change from 1^st^ to 13^th^ in solid-red line, and from 7^th^ to 1^st^ in dashed-yellow line, Fig. 3, lower panels). This population-wise difference in effect of acidification and freshening on male performance was seen clearly in correlations of performance rank among the treatments (Table 2). Rank order of performance in males from the recently invaded population under control conditions was significantly correlated with that under all combinations of acidification and freshening (*p* ≤ 0.05, Table 2), whereas performance rank in males from the established population were only significantly correlated between control conditions and acidification at 33 PSU. No negative rank-order correlations were observed.

**Table 2:** Correlation of sperm performance (SAAS rank) in the ambient (control) treatment (606 µatm CO_2_ × 33 PSU) with performance under acidification and freshening (Spearman’s rank correlation coefficient *ρ*).

Freshening (salinity, PSU)	33		23			13		
Acidification (*p*CO_2_, µatm)	925	1250	606	925	1250	606	925	1250
Invasive, Sweden	**0.80**	**0.61**	**0.74**	**0.72**	**0.70**	**0.70**	**0.64**	**0.56**
Established, Guernsey	**0.60**	**0.66**	0.20	0.27	0.36	0.35	0.16	0.27

*ρ* values with *p* ≤ 0.05 shown in bold.

## Discussion

Our finding that different populations, and individuals, of oysters showed distinctly different responses to ocean acidification and freshening could have pervasive implications for this species under marine climate change. We measured sperm motility using a metric (SAAS^27^) that integrates sperm swimming speed and percent motility, two key determinants of fertilisation success in free-spawning organisms^31,32^. Consequently, the large changes in motility we observed under acidification and freshening imply that these drivers could cause correspondingly large shifts in fertilisation success for individuals^33^. If sperm responses to acidification and freshening are heritable, these findings indicate there is substantial, and population-dependent, potential for selection and adaptation of this species under future climate change.

At the population level, clear patterns of response in oyster sperm motility emerged. Average sperm motility for the individuals we sampled from an established population increased with acidification, but decreased with freshening, such that these two drivers cancelled each other out at low salinity and high *p*CO_2_; a combination that is likely to manifest regionally in the future. In contrast, sperm motility of oysters sampled from an invasive population was unaffected by acidification (a result also obtained previously^20^), but decreased with freshening irrespective of *p*CO_2_. Such population-level differences in the effects of acidification have also been reported in another oyster (i.e. Sydney rock oyster, *Saccostrea glomerata*^3^). It is possible the different responses we observed were driven by parental environmental history^34^ and/or local adaptation^35^, although in that context it is surprising that oysters from the invasive population, which has recently undergone strong selection for tolerance to low salinities^28^, generally responded more negatively to freshening than those oysters from the established population that has lived many decades in ≈34 PSU. Equally, it is possible that some form of bottleneck or founder event has resulted in reduced genetic diversity of the invasive population (relative to the established population)^36,37^. Irrespective of the mechanism, such population-specific findings highlight the difficulties – and dangers – of forecasting species responses to climate drivers based on investigations of only one population.

Each of the populations considered showed substantial inter-individual variation in sperm motility responses. While some oysters responded negatively to treatments, others showed no response, with some responding positively. Such patterns of strong inter-individual variability in responses can reflect differences among parental genotypes and environments that then influence gametes exposed to different environmental conditions^38,39^. Alternatively, differences among individuals could result from non-genetic paternal effects such as differential gamete maturity. Variability between individuals has been reported previously in the context of organism responses to ocean acidification^7,8,20,33,40^. We are, however, unaware of any other study that has investigated the effects of multiple climate change-related drivers on individual sperm performance (but for the combined effects of ocean acidification and a toxicant – i.e. copper – see^41^). The growing body of literature showing that inter-individual variability in responses may be common^42^, argues for shifting focus away from understanding “mean” responses and toward investigating changes in variance and (hence) the potential for differential individual success in a future ocean.

Importantly, we found that variability of individual responses itself varied among populations and treatments. Freshening led to a clear increase in inter-individual variance in sperm motility (SAAS), particularly in the established population. In contrast, acidification had no effect on response variance, which – within each salinity – remained more or less constant in both populations. As noted earlier, shifts in variance of these populations may have been influenced by the environment of the parental organism. That is, the narrower response variance seen in oysters from the invasive population (Fig. 2) could reflect the strong salinity-driven selection this invasive population has recently experienced^28^, potentially reducing phenotypic variance. Alternately, this could be due to bottleneck and/or founder events that caused reductions in genetic diversity of these populations^36,37^. Nonetheless, recent work has shown local selection in this species can be pervasive^35^. Despite differences in magnitude of responses, the general pattern of increased variance under freshening indicates that differences among males will be amplified, a pattern which could have implications for male competitiveness during broadcast spawning.

The relative performance of sperm from different males shifted under the combined effects of acidification and freshening. For the established population, males that performed well under control (ambient) conditions performed poorly when exposed to acidification and freshening (Fig. 3, Table 2). This is the first such result for multiple climate drivers and extends a recent observation that acidification can change the male fitness landscape in broadcast spawners, and that this response can be modified by a co-factor (specifically a heavy metal^5^). In the invasive population, however, the relative performance of male oysters from the invasive population showed no substantive shift under freshening and acidification: males that performed well under control conditions continued to perform well under freshening and acidification (Fig. 3, Table 2). Given that sperm motility is a key determinant of fertilisation success^31,32^ – and hence adaptive capacity – continuing to explore how this feature can be modified by climate drivers and by the environmental history of a given population should be a focus of future research.

In conclusion, despite the burgeoning number of studies reporting effects of ocean acidification on the early life history of marine organisms, few have considered the importance of response variability, particularly for multiple climate drivers. Here, we show that the influence of ocean acidification on sperm motility and performance rank of individual male oysters can be strongly modified when combined with freshening, an effect that differs between populations and among individuals. This finding shows that quantifying inter- and intra-population response variability will be essential if we are to reliably project species’ responses to future climate drivers.

## Methods

### Experimental design

We investigated responses of sperm from multiple male oysters from two populations (*n* = 14 individuals from each population), to three levels of *p*CO_2_ and three levels of salinity in a fully-factorial design.

### Experimental organisms

Pacific oysters were collected in the summer of 2015 from an “established” population, which has been exposed to oceanic seawater for over 60 years at Guernsey Sea Farms, British Channel Islands. This population is open to some immigration, and is genetically representative of farmed populations from which feral populations have established and spread. These oysters were conditioned to reproductive maturity by Guernsey Sea Farms Ltd, Vale, Channel Islands, and shipped to the Tjärnö Marine Laboratory, Sweden, immediately prior to experimentation. Oysters were also collected haphazardly from a recent “invasive” population (established ≤10 years prior to this study^28^) in western Sweden. This environment is characterised by highly variable salinity and pH (13–30 PSU, 7.7–8.1 pH)^43^. Following collection, oysters from the invasive population were conditioned to reproductive maturity by Ostrea Sverige AB, South Koster, Sweden, with the same methods used by Guernsey Sea Farms Ltd. Conditioned oysters were held in temperature-controlled flowing seawater at the Tjärnö Marine Laboratory at 33 PSU and 17 °C (just below the temperature that elicits spawning) until experiments were run.

### Experimental treatments

*p*CO_2_ treatment levels were selected to reflect ambient inflowing seawater *p*CO_2_ at the Tjärnö Marine Laboratory (606 µatm), late-century atmospheric *p*CO_2_ (925 µatm), and end-of-century atmospheric *p*CO_2_ (1250 µatm) under prevalent global climate change scenarios^44^. Treatment levels were achieved by bubbling CO_2_-air mixtures into the treatment water until a given target pH_NBS_ was obtained. pH_NBS_ set-points equivalent to treatment *p*CO_2_ levels were determined by calibrating pH_NBS_ against inflowing seawater equilibrated for 24 h with custom mixed gases of the relevant CO_2_ concentration (AGA, Sweden AB). The response slope of the pH meter was calibrated daily using NBS buffers. Total alkalinity was estimated from salinity using a long-term salinity:alkalinity relationship for this location (r = 0.94)^43^. This method has been shown to generate very low uncertainties in estimates of carbonate system parameters (uncertainties ≤±0.006 pH_NBS_ and ±0.08 Ω_*Ar*_) (for more details see^45^). *p*CO_2_ and saturation states for calcite (Ω_Ca_) and aragonite (Ω_Ar_) were calculated using CO2calc^46^, with constants from Mehrbach *et al*.^47^ adjusted by Dickson and Millero^48^ (Table 3).

**Table 3:** Seawater chemistry of experimental treatments of ocean acidification (*p*CO_2_) and freshening (salinity).

Treatment (nominal salinity, *p*CO_2_)	Salinity (PSU)	pH	Temp (°C)	A_T_ (µmol kg^−1^)	*p*CO_2_ (µatm)	Ω_Ca_	Ω_Ar_
33, 606	32.9 (<0.1)	8.02 (<0.01)	20.0 (0.2)	2261	620	3.23	2.09
33, 925	32.9 (<0.1)	7.86 (<0.01)	20.0 (0.2)	2261	938	2.34	1.51
33, 1250	33.0 (<0.1)	7.74 (<0.01)	20.0 (0.2)	2265	1270	1.82	1.18
23, 606	23.0 (<0.1)	8.03 (<0.01)	20.0 (0.2)	2004	604	2.44	1.52
23, 925	23.0 (<0.1)	7.86 (<0.01)	20.0 (0.2)	2004	927	1.71	1.07
23, 1250	23.0 (<0.1)	7.74 (<0.01)	20.0 (0.2)	2004	1245	1.32	0.82
13, 606	13.0 (<0.1)	8.04 (<0.01)	20.0 (0.2)	1743	596	1.73	1.01
13, 925	13.0 (<0.1)	7.86 (<0.01)	20.0 (0.2)	1743	927	1.17	0.69
13, 1250	13.0 (<0.1)	7.73 (<0.01)	20.0 (0.2)	1743	1268	0.88	0.52

Direct measures of salinity, pH, and temperature (Temp) were made on each experimental day (*n* = 9 measures). Total alkalinity (A_T_) was estimated from an established salinity:alkalinity relationship for the sea for this region (r = 0.94). Partial pressure of CO_2_ (*p*CO_2_), and saturation states for calcite (Ω_Ca_) and aragonite (Ω_Ar_) using CO2calc (details in text). Data are mean (SE).

Salinity treatment levels were chosen to reflect the full-strength seawater typical of Guernsey (33 PSU), local sea surface salinity at Tjärnö, western Sweden (23 PSU), and sea-surface salinity in south-western Sweden (13 PSU), toward which the invasive oysters are spreading^28^. Salinity was manipulated by diluting filtered sea water with filtered fresh water. Treatment levels were verified using a conductivity meter (WTW, Cond 3210, Germany) calibrated against laboratory salinity standards (Table 3).

### Response variable: sperm motility (SAAS)

Sperm motility for each replicate male (*n* = 14 per population), was quantified by measuring Sperm Accumulated Against Surface (SAAS)^27^ under all combinations of *p*CO_2_ and salinity. For each male oyster, concentrated sperm were extracted directly from the testis using a Pasteur pipette inserted through a hole drilled in the shell above the gonad. Previous work has shown that any non-motile sperm released by this technique will have had minimal impact on the results^27^. Namely, SAAS is almost exclusively caused by actively swimming sperm, and the sinking rate of dead/inactive sperm is so slow that it does not bias results obtained using the technique. Concentrated sperm were stored in an Eppendorf tube on ice. Sperm concentration in each suspension was verified by hemocytometer counts of an aliquot of sperm that had been immobilised and stained with Lugol’s solution. For each male, independent sub-samples of sperm from the stock suspension were diluted into each of three replicate wells of a multi-well plate containing treatment seawater (three replicates per treatment combination and male). Volumes of sperm transferred were adjusted to ensure a final concentration of 2 × 10^6^ sperm ml^−1^ in the treatments. Sperm were exposed to the treatment seawater for 10 min, after which 1.5 ml of the suspension was pipetted to a new multi-well plate from which the pattern of accumulation of sperm on the bottom surface of the well over time was quantified. Sperm counts were determined using a phase-contrast inverted microscope (Leica, DMIL, Germany) equipped with a digital camera (PixeLINK, PL-D725CU, Canada). To quantify accumulation, still images of different (central) areas of the lower surface of the wells were taken 10 min after the addition of the sperm suspension to the well (*n = *3 images per well). Digital images were post-processed and the number of sperm that had accumulated at the lower surface of the well (SAAS) counted manually.

### Statistical analysis

The response of sperm motility (SAAS) to *p*CO_2_ and salinity was determined by calculating logarithmic response ratios, LnRR = log_e_ ([response in treatment]/[response in control]), for each treatment combination and male, using the 606 µatm CO_2_ × 33 PSU treatment as the control. Unlike raw ratio data, log_e_ response ratios are approximately normally distributed^49^, and therefore we also calculated mean LnRR ± 95% CI. We determined the statistical significance of sperm responses to the treatments using a three-way permutational analysis of variance (PERMANOVA) using the PRIMER package^50^ with population, *p*CO_2_, and salinity as fixed factors, and individuals as a random factor (*n* = 14 per population, 3 replicates per male) nested within population. To reduce heterogeneity of variances, data were square root transformed before analysis.

## Data Availability

The datasets generated during and/or analysed during the current study are available from the corresponding author on reasonable request.

## References

[1] Kroeker KJ (2013). Impacts of ocean acidification on marine organisms: quantifying sensitivities and interaction with warming. Global Change Biology.

[2] Waldbusser G, Bergschneider H, Green M (2010). Size-dependent pH effect on calcification in post-larval hard clam Mercenaria spp. Marine Ecology Progress Series.

[3] Parker LM, Ross PM, O’Connor WA (2011). Populations of the Sydney rock oyster, Saccostrea glomerata, vary in response to ocean acidification. Marine Biology.

[4] Walther K, Sartoris FJ, Pörtner HO (2011). Impacts of temperature and acidification on larval calcium incorporation of the spider crab Hyas araneus from different latitudes (54° vs. 79°N). Marine Biology.

[5] Campbell AL, Levitan DR, Hosken DJ, Lewis C (2016). Ocean acidification changes the male fitness landscape. Scientific Reports.

[6] Pistevos JCA, Calosi P, Widdicombe S, Bishop JDD (2011). Will variation among genetic individuals influence species responses to global climate change?. Oikos.

[7] Schlegel P, Havenhand JN, Obadia N, Williamson JE (2014). Sperm swimming in the polychaete Galeolaria caespitosa shows substantial inter-individual variability in response to future ocean acidification. Marine Pollution Bulletin.

[8] Schlegel P, Havenhand JN, Gillings MR, Williamson JE (2012). Individual variability in reproductive success determines winners and losers under ocean acidification: a case study with sea urchins. PLOS ONE.

[9] Foo SA, Dworjanyn SA, Poore AGB, Byrne M (2012). Adaptive capacity of the habitat modifying sea urchin Centrostephanus rodgersii to ocean warming and ocean acidification: performance of early embryos. PLOS ONE.

[10] Reed DH, Frankham R (2003). Correlation between fitness and genetic diversity. Conservation Biology.

[11] Frankham R (2005). Conservation biology: ecosystem recovery enhanced by genotypic diversity. Heredity.

[12] Levitan DR (2008). Gamete traits influence the variance in reproductive success, the intensity of sexual selection, and the outcome of sexual conflict among congeneric sea urchins. Evolution.

[13] Levitan DR (1996). Effects of gamete traits on fertilization in the sea and the evolution of sexual dimorphism. Nature.

[14] Graham H (2016). Sperm motility and fertilisation success in an acidified and hypoxic environment. ICES Journal of Marine Science.

[15] Gazeau F (2013). Impacts of ocean acidification on marine shelled molluscs. Marine Biology.

[16] Schlegel P, Binet MT, Havenhand JN, Doyle CJ, Williamson JE (2015). Ocean acidification impacts on sperm mitochondrial membrane potential bring sperm swimming behaviour near its tipping point. The Journal of Experimental Biology.

[17] Havenhand JN, Buttler F-R, Thorndyke MC, Williamson JE (2008). Near-future levels of ocean acidification reduce fertilization success in a sea urchin. Current Biology.

[18] Morita M (2010). Ocean acidification reduces sperm flagellar motility in broadcast spawning reef invertebrates. Zygote.

[19] Vihtakari M (2013). Effects of ocean acidification and warming on sperm activity and early life stages of the Mediterranean mussel (Mytilus galloprovincialis). Water.

[20] Havenhand JN, Schlegel P (2009). Near-future levels of ocean acidification do not affect sperm motility and fertilization kinetics in the oyster Crassostrea gigas. Biogeosciences.

[21] Caldwell GS (2011). Ocean acidification takes sperm back in time. *Invertebrate Reproduction &*. Development.

[22] Boyd PW (2018). Experimental strategies to assess the biological ramifications of multiple drivers of global ocean change - a review. Global Change Biology.

[23] Hoegh-Guldberg O, Bruno JF (2010). The impact of climate change on the world’s marine ecosystems. Science.

[24] Doney SC (2012). Climate change impacts on marine ecosystems. Annual Review of Marine Science.

[25] Shi P, Sun S, Gong D, Zhou T (2016). World Regionalization of Climate Change (1961–2010). International Journal of Disaster Risk Science.

[26] Litvak MK, Trippel EA (1998). Sperm motility patterns of Atlantic cod (Gadus morhua) in relation to salinity: effects of ovarian fluid and egg presence. Canadian Journal of Fisheries and Aquatic Sciences.

[27] Falkenberg LJ, Havenhand JN, Styan CA (2016). Sperm Accumulated Against Surface: a novel alternative bioassay for environmental monitoring. Marine Environmental Research.

[28] Wrange A-L (2010). Massive settlements of the Pacific oyster, Crassostrea gigas, in Scandinavia. Biological Invasions.

[29] Parker LM, Ross PM, O’Connor WA (2010). Comparing the effect of elevated pCO2 and temperature on the fertilization and early development of two species of oysters. Marine Biology.

[30] Muranaka MS, Lannan JE (1984). Broodstock management of Crassostrea gigas: environmental influences on broodstock conditioning. Aquaculture.

[31] Styan CA (1998). Polyspermy, egg size, and the fertilization kinetics of free-spawning marine invertebrates. The American Naturalist.

[32] Vogel H, Czihak G, Chang P, Wolf W (1982). Fertilization kinetics of sea urchin eggs. Mathematical Biosciences.

[33] Vihtakari M, Havenhand J, Renaud PE, Hendriks IE (2016). Variable individual- and population- level responses to ocean acidification. Frontiers in Marine Science.

[34] Hofmann GE (2014). Exploring local adaptation and the ocean acidification seascape - studies in the California Current Large Marine Ecosystem. Biogeosciences.

[35] Li L (2018). Divergence and plasticity shape adaptive potential of the Pacific oyster. *Nature Ecology &*. Evolution.

[36] Rohfritsch A (2013). Population genomics shed light on the demographic and adaptive histories of European invasion in the Pacific oyster, *Crassostrea gigas*. Evolutionary Applications.

[37] Anglès d’Auriac MB (2017). Rapid expansion of the invasive oyster Crassostrea gigas at its northern distribution limit in Europe: Naturally dispersed or introduced?. PLOS ONE.

[38] Crean AJ, Dwyer JM, Marshall DJ (2013). Adaptive paternal effects? Experimental evidence that the paternal environment affects offspring performance. Ecology.

[39] Jensen N, Allen RM, Marshall DJ (2014). Adaptive maternal and paternal effects: gamete plasticity in response to parental stress. Functional Ecology.

[40] Frommel AY, Stiebens V, Clemmesen C, Havenhand J (2010). Effect of ocean acidification on marine fish sperm (Baltic cod: Gadus morhua). Biogeosciences.

[41] Campbell AL, Mangan S, Ellis RP, Lewis C (2014). Ocean acidification increases copper toxicity to the early life history stages of the polychaete Arenicola marina in artificial seawater. Environmental Science & Technology.

[42] Calosi P (2013). Multiple physiological responses to multiple environmental challenges: an individual approach. Integrative and Comparative Biology.

[43] SMHI. Swedish Oceanographic Data Centre. Available at, http://www.smhi.se/oceanografi/oce_info_data/SODC/download_en.htm (2011).

[44] Collins, M. *et al*. In *Climate Change 2013: The Physical Science Basis. Contribution of Working Group I to the Fifth Assessment Report of the Intergovernmental Panel on Climate Change* (IPCC, 2013).

[45] Eriander L, Wrange A-L, Havenhand JN (2016). Simulated diurnal pH fluctuations radically increase variance in—but not the mean of—growth in the barnacle Balanus improvisus. ICES Journal of Marine Science.

[46] Robbins, L. L., Hansen, M. E., Kleypas, J. A. & Meylan, S. C. CO2calc: a user-friendly seawater carbon calculator for Windows, Mac OS X, and iOS (iPhone). *Open-File Report*, 10.3133/OFR20101280 (2010).

[47] Mehrbach C, Culberson CH, Hawley JE, Pytkowicx RM (1973). Measurement of the apparent dissociation constants of carbonic acid in seawater at atmpospheric pressure. Limnology and Oceanography.

[48] Dickson AG, Millero FJ (1987). A comparison of the equilibrium constants for the dissociation of carbonic acid in seawater media. Deep Sea Research Part A. Oceanographic Research Papers.

[49] Nakagawa S, Cuthill IC (2007). Effect size, confidence interval and statistical significance: a practical guide for biologists. Biological Reviews.

[50] Anderson MJ (2001). A new method for non-parametric multivariate analysis of variance. Austral Ecology.

